# Supplementary material for: Phenotype-specific differences in polygenicity and effect size distribution across functional annotation categories revealed by AI-MiXeR
Source: Bioinformatics. 2020 Jun 15;36(18):4749–56. doi: 10.1093/bioinformatics/btaa568 (PMC7750998; doi:10.1093/bioinformatics/btaa568)
Supplement: btaa568_supplementary_data [file btaa568_supplementary_data.zip › aimixer_supplementary_material_revised.docx]

# Revealing phenotype-specific differences in polygenicity and effect size distribution across functional annotation categories with AI-MiXeR

# Supplementary material

## Tables

| **Phenotype** | $\boldsymbol{\pi}_{\boldsymbol{non-exonic}}$ | $\boldsymbol{\pi}_{\boldsymbol{exonic}}$ | $\boldsymbol{\sigma}_{\boldsymbol{non-exonic}}^{\boldsymbol{2}}$ | $\boldsymbol{\sigma}_{\boldsymbol{exonic}}^{\boldsymbol{2}}$ | $\boldsymbol{\sigma}_{\boldsymbol{0}}^{\boldsymbol{2}}$ | $\boldsymbol{N}_{\boldsymbol{non-exonic}}$ | $\boldsymbol{N}_{\boldsymbol{exonic}}$ | $\boldsymbol{h}_{\boldsymbol{total}}^{\boldsymbol{2}}$ | $\boldsymbol{h}_{\boldsymbol{non-exonic}}^{\boldsymbol{2}}$ | $\boldsymbol{h}_{\boldsymbol{exonic}}^{\boldsymbol{2}}$ |
| --- | --- | --- | --- | --- | --- | --- | --- | --- | --- | --- |
| SCZ | 3.11E-03  (2.81E-03,3.37E-03) | 2.24E-02  (1.86E-02,2.79E-02) | 5.30E-05  (4.87E-05,5.80E-05) | 1.04E-04  (8.27E-05,1.26E-04) | 1.13  (1.12,1.13) | 30610  (27709,33163) | 3384  (2817,4227) | 4.01E-01  (3.97E-01,4.08E-01) | 3.37E-01  (3.29E-01,3.42E-01) | 6.46E-02  (6.14E-02,6.92E-02) |
| BD | 2.77E-03  (2.44E-03,3.21E-03) | 1.83E-02  (1.54E-02,2.08E-02) | 4.05E-05  (3.53E-05,4.60E-05) | 1.17E-04  (1.01E-04,1.37E-04) | 1.06  (1.06,1.07) | 27322  (24031,31576) | 2767  (2336,3143) | 2.89E-01  (2.83E-01,2.94E-01) | 2.29E-01  (2.23E-01,2.36E-01) | 5.99E-02  (5.56E-02,6.35E-02) |
| ADHD | 1.47E-03  (1.30E-03,1.63E-03) | 3.86E-05  (1.06E-05,8.14E-05) | 5.69E-05  (5.29E-05,6.12E-05) | 1.65E-03  (8.72E-04,3.29E-03) | 1.11  (1.11,1.12) | 14426  (12769,16000) | 6  (2,12) | 1.72E-01  (1.63E-01,1.77E-01) | 1.70E-01  (1.62E-01,1.76E-01) | 1.71E-03  (4.28E-04,3.10E-03) |
| COG | 2.72E-03  (2.56E-03,2.88E-03) | 8.93E-03  (6.83E-03,1.11E-02) | 2.54E-05  (2.40E-05,2.67E-05) | 6.96E-05  (5.77E-05,8.93E-05) | 1.22  (1.21,1.23) | 26747  (25225,28359) | 1352  (1034,1676) | 1.58E-01  (1.56E-01,1.61E-01) | 1.41E-01  (1.39E-01,1.44E-01) | 1.73E-02  (1.59E-02,1.91E-02) |
| EA | 4.86E-03  (4.67E-03,5.20E-03) | 1.78E-03  (1.32E-03,2.17E-03) | 9.77E-06  (9.11E-06,1.01E-05) | 1.24E-04  (1.01E-04,1.51E-04) | 1.20  (1.19,1.20) | 47809  (45961,51168) | 270  (200,328) | 1.03E-01  (1.02E-01,1.04E-01) | 9.70E-02  (9.60E-02,9.83E-02) | 6.19E-03  (5.64E-03,7.11E-03) |
| T2D | 8.07E-05  (6.53E-05,9.30E-05) | 5.37E-02  (4.87E-02,6.25E-02) | 4.37E-04  (3.87E-04,5.52E-04) | 3.98E-05  (3.40E-05,4.41E-05) | 1.18  (1.18,1.19) | 795  (643,915) | 8134  (7368,9465) | 1.32E-01  (1.27E-01,1.36E-01) | 7.19E-02  (6.68E-02,7.50E-02) | 6.01E-02  (5.83E-02,6.29E-02) |
| IBD | 2.73E-05  (2.26E-05,3.28E-05) | 1.84E-02  (1.69E-02,1.99E-02) | 2.12E-03  (1.75E-03,2.47E-03) | 2.61E-04  (2.21E-04,2.95E-04) | 1.20  (1.19,1.20) | 269  (222,323) | 2788  (2555,3006) | 2.52E-01  (2.41E-01,2.62E-01) | 1.17E-01  (1.11E-01,1.27E-01) | 1.35E-01  (1.22E-01,1.43E-01) |
| LDL | 1.47E-04  (5.18E-05,2.06E-04) | 1.77E-03  (1.53E-03,2.52E-03) | 1.42E-04  (1.02E-04,3.07E-04) | 1.99E-03  (1.29E-03,2.35E-03) | 0.96  (0.95,0.98) | 1448  (510,2027) | 268  (232,381) | 1.39E-01  (1.24E-01,1.47E-01) | 4.12E-02  (3.26E-02,4.53E-02) | 9.82E-02  (9.11E-02,1.05E-01) |
| BMI | 3.64E-03  (3.50E-03,3.95E-03) | 4.05E-03  (3.39E-03,4.89E-03) | 2.33E-05  (2.16E-05,2.43E-05) | 1.67E-04  (1.42E-04,2.06E-04) | 1.61  (1.59,1.62) | 35834  (34414,38871) | 613  (513,741) | 1.93E-01  (1.91E-01,1.95E-01) | 1.74E-01  (1.72E-01,1.75E-01) | 1.90E-02  (1.79E-02,1.98E-02) |
| HEIGHT | 6.06E-04  (5.79E-04,6.27E-04) | 6.32E-02  (5.94E-02,6.96E-02) | 2.70E-04  (2.56E-04,2.83E-04) | 9.26E-05  (8.11E-05,9.94E-05) | 2.41  (2.38,2.44) | 5966  (5699,6169) | 9562  (8989,10532) | 4.99E-01  (4.90E-01,5.03E-01) | 3.34E-01  (3.27E-01,3.42E-01) | 1.65E-01  (1.59E-01,1.70E-01) |
| WHR | 4.17E-04  (3.76E-04,4.54E-04) | 6.97E-02  (4.68E-02,3.13E-01) | 4.92E-05  (4.56E-05,5.31E-05) | 8.01E-06  (1.56E-06,1.13E-05) | 0.94  (0.94,0.95) | 4107  (3697,4468) | 10556  (7082,47316) | 5.64E-02  (5.50E-02,5.79E-02) | 4.19E-02  (4.05E-02,4.36E-02) | 1.45E-02  (1.38E-02,1.52E-02) |
| **Supplementary Table S1**. Estimates of proportion and number of non-null variants, their average effect sizes and heritability in exonic and non-exonic annotation categories.  The numbers represent mean (min, max) values taken across 50 independent optimization runs with 10^6^ randomly selected variants used in the cost function for optimization.  $N_{exonic}$ = number of non-null variants in the exonic category = ${\pi_{exonic}\times T}_{exonic}$, where $T_{exonic}$ is the total number of variants in the exonic category. and $N_{non-exonic}= {\pi_{non-exonic}\times T}_{non-exonic}$, in the LDSC template used in our study $T_{non-exonic} \cong70*T_{exonic}$ and $T_{non-exonic}+T_{exonic}\cong{10}^{7}$. Phenotypes: schizophrenia (SCZ), bipolar disorder (BD), attention deficit/hyperactivity disorder (ADHD), general cognitive ability (COG), educational attainment (EA), type 2 diabetes (T2D), inflammatory bowel disease (IBD), low-density lipoproteins (LDL), body mass index (BMI), height and waist-hip ratio (WHR). | | | | | | | | | | |

| **Phenotype** | **MiXeR** | | | **LDSC** | | |
| --- | --- | --- | --- | --- | --- | --- |
|  | $\boldsymbol{h}_{\boldsymbol{total}}^{\boldsymbol{2}}$ | **Enrichment non-exonic** | **Enrichment exonic** | $\boldsymbol{h}_{\boldsymbol{total}}^{\boldsymbol{2}}$ | **Enrichment non-exonic** | **Enrichment exonic** |
| SCZ | 0.40  (4.73E-03,6.33E-03) | 0.85  (1.05E-02,8.92E-03) | 10.63  (5.80E-01,6.81E-01) | 0.37  (1.71E-02) | 0.91  (1.65E-02) | 7.64  (1.20E+00) |
| BD | 0.29  (5.58E-03,5.13E-03) | 0.80  (1.25E-02,1.23E-02) | 13.69  (7.97E-01,8.14E-01) | 0.27  (1.59E-02) | 0.86  (2.61E-02) | 11.19  (1.89E+00) |
| ADHD | 0.17  (8.81E-03,4.85E-03) | 1.01  (8.32E-03,7.64E-03) | 0.66  (4.97E-01,5.41E-01) | 0.19  (1.46E-02) | 1.03  (2.67E-02) | -1.11  (1.94E+00) |
| COG | 0.16  (2.23E-03,2.37E-03) | 0.90  (9.77E-03,9.86E-03) | 7.23  (6.41E-01,6.35E-01) | 0.16  (6.60E-03) | 0.94  (1.39E-02) | 5.69  (1.01E+00) |
| EA | 0.10  (1.13E-03,1.14E-03) | 0.95  (8.38E-03,5.31E-03) | 3.96  (3.46E-01,5.45E-01) | 0.10  (2.90E-03) | 0.97  (1.26E-02) | 2.97  (9.13E-01) |
| T2D | 0.13  (4.55E-03,4.01E-03) | 0.55  (2.06E-02,1.76E-02) | 30.06  (1.14E+00,1.34E+00) | 0.13  (9.50E-03) | 0.67  (4.25E-02) | 25.29  (3.09E+00) |
| IBD | 0.25  (1.15E-02,9.46E-03) | 0.47  (2.27E-02,2.66E-02) | 35.30  (1.73E+00,1.47E+00) | 0.23  (2.09E-02) | 0.55  (5.99E-02) | 33.57  (4.35E+00) |
| LDL | 0.14  (1.57E-02,7.84E-03) | 0.30  (3.28E-02,2.53E-02) | 46.51  (1.64E+00,2.13E+00) | 0.14  (1.73E-02) | 0.50  (9.13E-02) | 37.44  (6.63E+00) |
| BMI | 0.19  (1.68E-03,1.96E-03) | 0.92  (4.09E-03,5.72E-03) | 6.50  (3.72E-01,2.66E-01) | 0.19  (6.60E-03) | 0.92  (1.27E-02) | 6.56  (9.19E-01) |
| HEIGHT | 0.50  (8.81E-03,4.10E-03) | 0.68  (9.50E-03,1.29E-02) | 21.77  (8.36E-01,6.18E-01) | 0.47  (2.07E-02) | 0.77  (2.61E-02) | 17.37  (1.90E+00) |
| WHR | 0.06  (1.44E-03,1.48E-03) | 0.75  (1.20E-02,1.36E-02) | 16.96  (8.86E-01,7.83E-01) | 0.07  (5.50E-03) | 0.84  (3.10E-02) | 12.42  (2.25E+00) |
| **Supplementary Table S2**. Comparison of partitioned heritability estimates from AI-MiXeR and LDSC.  For AI-MiXeR, confidence intervals are presented as (mean – min, max - mean) where mean, min and max are taken across 50 runs of cost function optimization with 10^6^ randomly selected variants. For LDSC, standard errors are given in brackets. | | | | | | |

**Supplementary Table S3**. Results for all 810 simulation scenarios (in separate Excel file).

## Figures

| 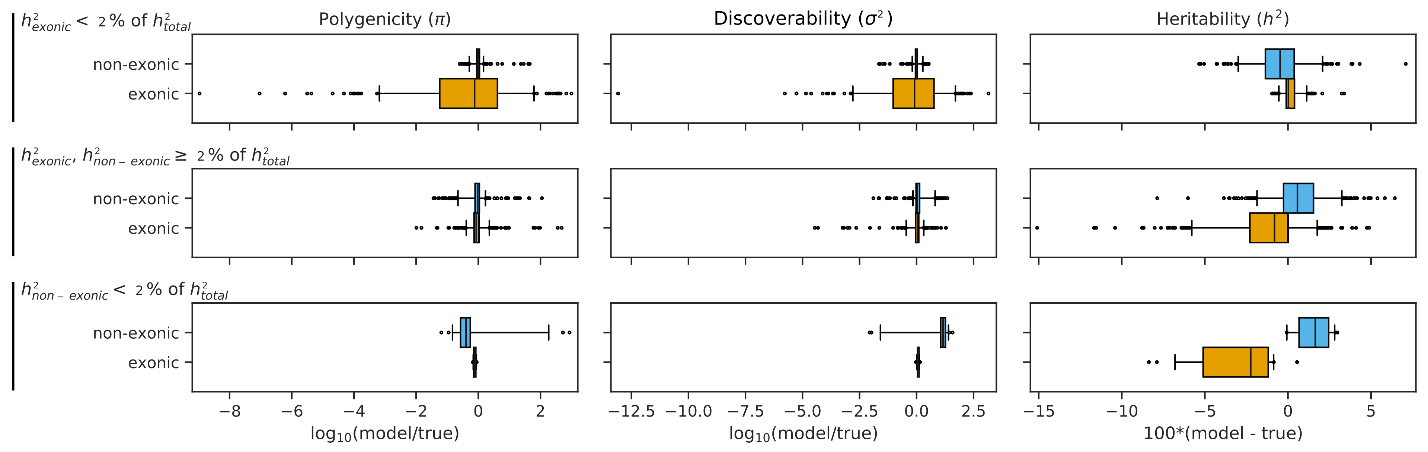 |
| --- |
| **Supplementary Figure S1.** Performance of the model on synthetic GWAS data.  Three different scenarios: $h_{exonic}^{2}$ < 2% of $h_{total}^{2}$, 300 tests (top row), both $h_{exonic}^{2}$ and $h_{non-exonic}^{2}$ >= 2% of $h_{total}^{2}$, 480 tests (middle row), and $h_{non-exonic}^{2}$ < 2% $h_{total}^{2}$, 30 tests (bottom row).  The color coding defines the functional annotation category (blue: non-exonic, orange: exonic).  For polygenicity (first column) and discoverability (second column), the X axis shows by how many orders of magnitudes the modeled parameter is greater than corresponding real parameter: log_10_(model/true). For heritability, the X axis shows the difference between modeled and true heritabilties (measured in percents, i.e. $0\leq h^{2}\leq100$). For all subplots, closer to 0corresponds to better performance of the model.  Boxes include 50% of all tests, whiskers show 95% confidence intervals, dots represent outliers. |

| 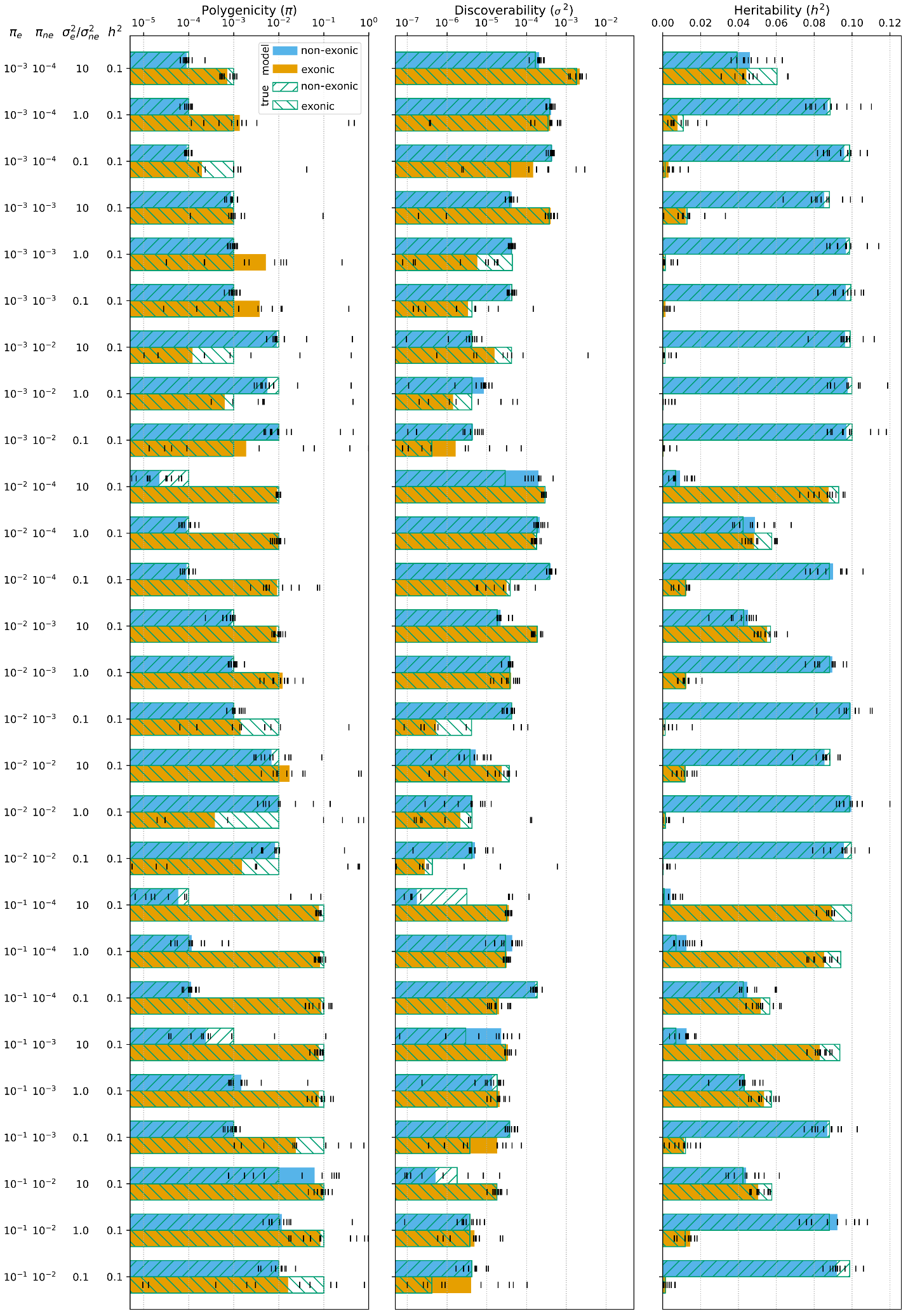 |
| --- |
| **Supplementary Figure S2.** Simulations with $h_{total}^{2}$ = $h_{exonic}^{2}$+$h_{non-exonic}^{2}$ = 0.1 and all combinations of $\pi_{exonic}$ = 10^-1^, 10^-2^, 10^-3^; $\pi_{non-exonic}$ = 10^-2^, 10^-3^, 10^-4^ and $\sigma_{exonic}^{2}$/$\sigma_{non-exonic}^{2}$ = 0.1, 1.0, 10.0$.$ |
| 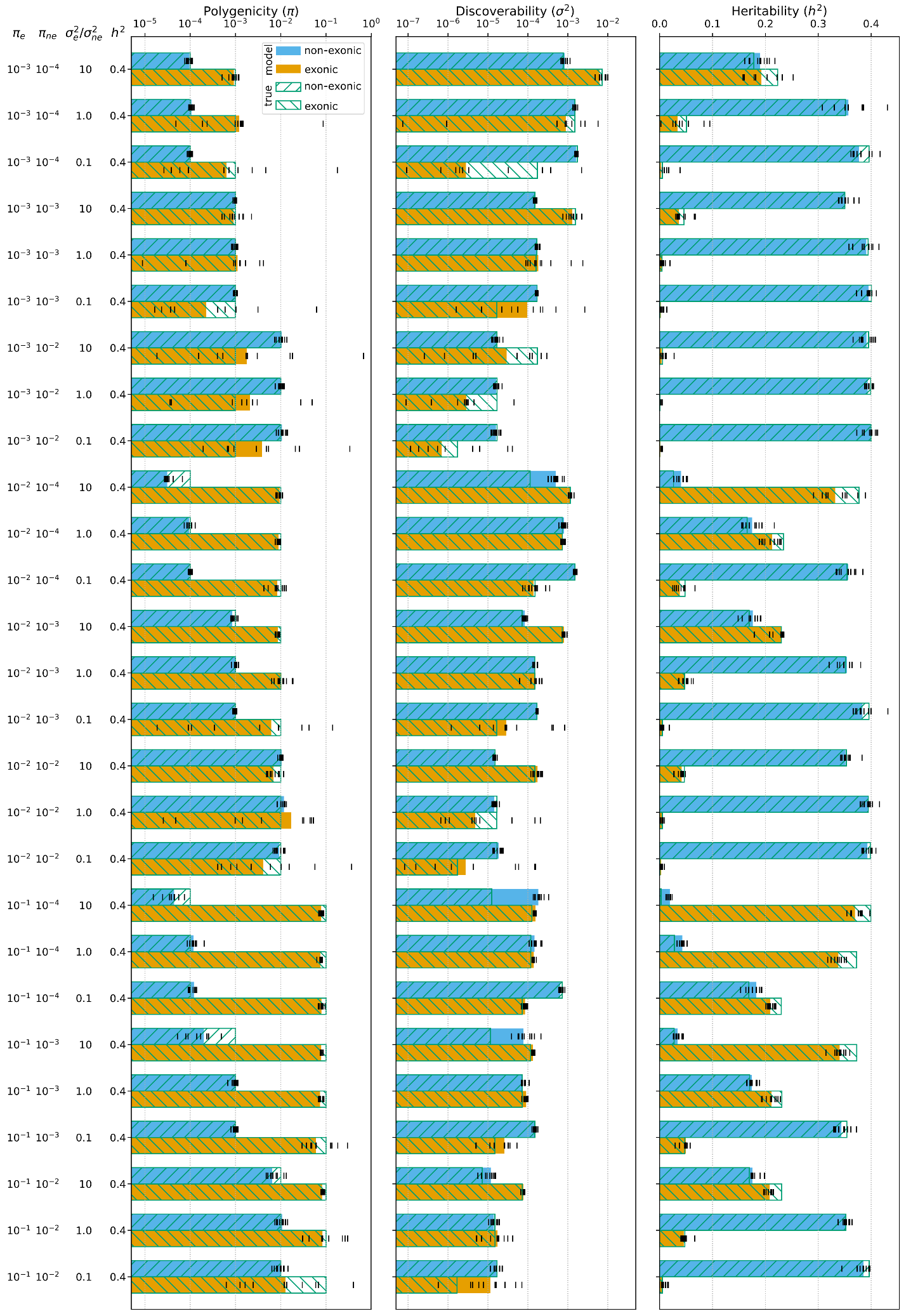 |
| **Supplementary Figure S3.** Simulations with $h_{total}^{2}$ = $h_{exonic}^{2}$+$h_{non-exonic}^{2}$ = 0.4 and all combinations of $\pi_{exonic}$ = 10^-1^, 10^-2^, 10^-3^; $\pi_{non-exonic}$ = 10^-2^, 10^-3^, 10^-4^ and $\sigma_{exonic}^{2}$/$\sigma_{non-exonic}^{2}$ = 0.1, 1.0, 10.0$.$ |
| 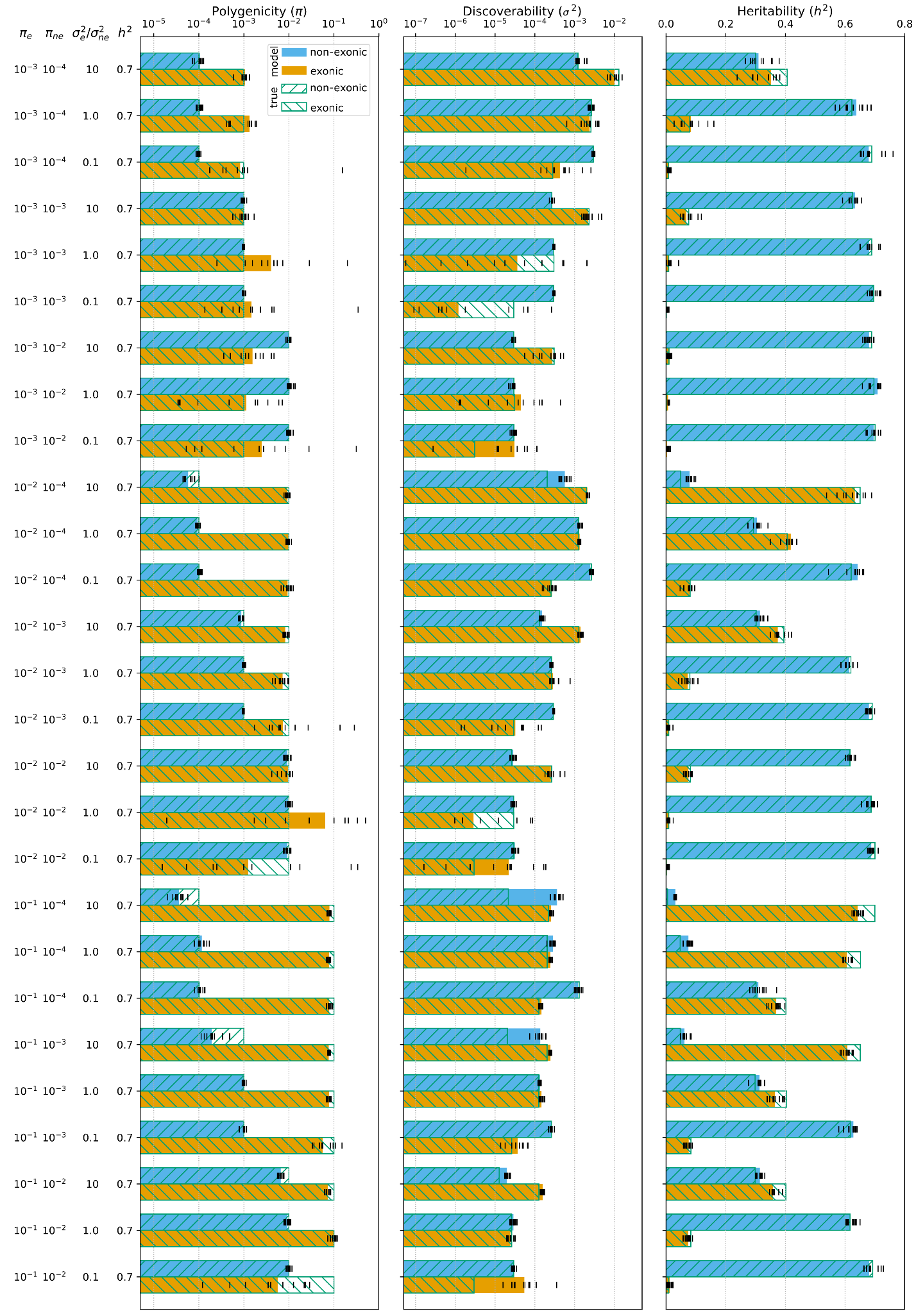 |
| **Supplementary Figure S4.** Simulations with $h_{total}^{2}$ = $h_{exonic}^{2}$+$h_{non-exonic}^{2}$ = 0.7 and all combinations of $\pi_{exonic}$ = 10^-1^, 10^-2^, 10^-3^; $\pi_{non-exonic}$ = 10^-2^, 10^-3^, 10^-4^ and $\sigma_{exonic}^{2}$/$\sigma_{non-exonic}^{2}$ = 0.1, 1.0, 10.0. |

## Exploratory analysis of exonic, intronic, promoter and enhancer regions

In this exploratory analysis we have evaluated six different annotation categories using the same two-way partitioning procedure as in the main analysis (Supplementary Table S4), where we optimized parameters for each annotation category and its complementary category separately. For each primary annotation category its complementary category represents all variants not included in the primary category.

| **Primary annotation category** | **Number of variants in** | |
| --- | --- | --- |
|  | **Primary category** | **Complementary category** |
| Exons | 151,398 | 9,845,833 |
| Exons + Promoters | 314,975 | 9,682,256 |
| Exons + Promoters + Enhancers | 1,667,126 | 8,330,105 |
| Exons + Introns | 3,173,386 | 6,823,845 |
| Exons + Introns + Promoters | 3,336,963 | 6,660,268 |
| Exons + Introns + Promoters + Enhancers | 4,689,114 | 5,308,117 |
| **Supplementary Table S4.** Functional annotation categories considered in the analysis. | | |

Like in the main analysis, the Exons category included all variants within exons (including 5’ and 3’ untranslated regions) of protein coding genes. Introns contained all variants within introns of protein coding genes. Promoters were defined as 2Kbp region upstream protein coding genes. Enhancers were defined as 48Kbp region upstream Promoters. If a variant was located in multiple sub-categories (for example when the promoter of one gene overlaps with the last exon of the upstream gene), it was assigned to a single sub-category according to the priority order: Exons > Promoters > Introns > Enhancers. We used the same variant template (containing 9,997,231 biallelic variants) as in the main analysis.

This exploratory analysis was limited to schizophrenia (SCZ) and type 2 diabetes (T2D) which demonstrated diverse patterns of effect size distributions in the main analysis.

The optimization setup was similar to the setup in the main analysis with one difference: we performed 15 (vs 50 in the main analysis) independent optimization runs for each phenotype and primary/complementary annotation category partition. Results are shown in Supplementary Figure S5.

Interestingly, consistently better likelihoods (smaller normalized costs) for SCZ are observed when the primary annotation category includes introns. In contrast, for T2D the best likelihood is obtained when the primary annotation category consists of exons and promoters, while adding introns reduces the likelihood significantly.

| 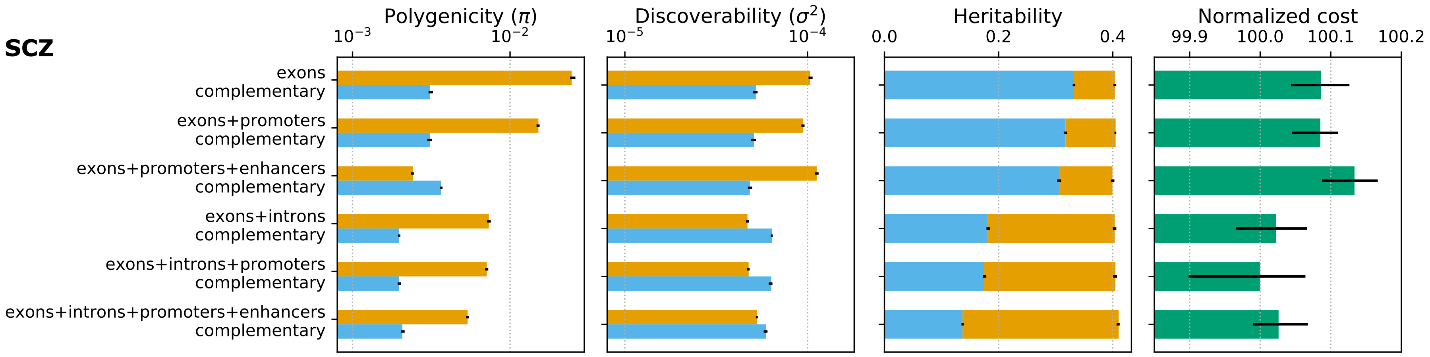 |
| --- |
| 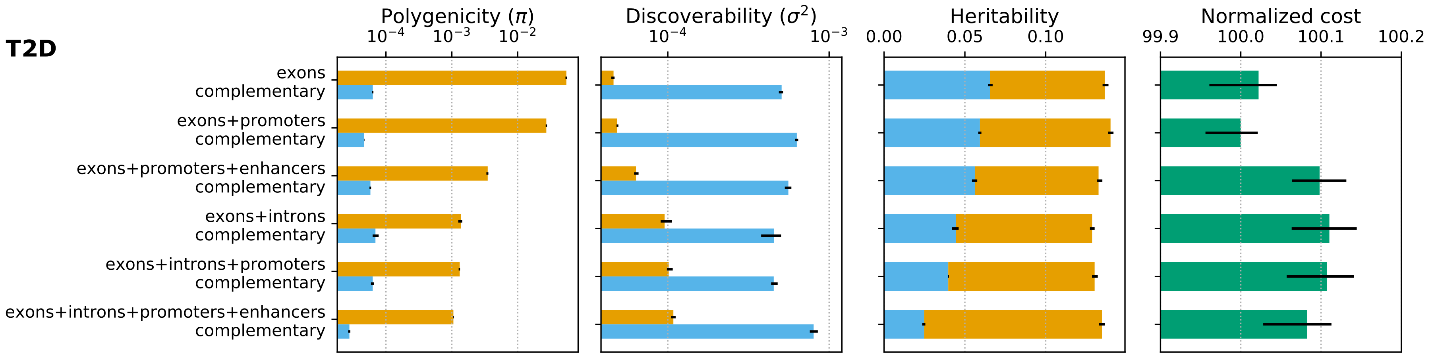 |
| **Supplementary Figure S5.** Estimated polygenicity (proportion of non-null variants), discoverability (variance of non-null effect sizes) and heritability of six primary annotation categories and corresponding complementary categories, and normalized cost for each primary/complementary partition.  The top panel shows schizophrenia (SCZ), the bottom one type 2 diabetes (T2D) results.  The orange bars represent the primary annotation category, the blue bars represent the corresponding complementary category.  A bar’s length shows the mean value of the parameter obtained from 15 independent optimization runs with 106 randomly selected variants used to maximize the likelihood of the observed GWAS z-scores. The black bars show the range (min, max) of such estimates.  Normalized cost = K*log(likelihood)/N, where N = 106 is the number of variants used for optimization, K is a scaling factor selected for each phenotype separately to make the minimum average (over 15 optimization runs) log(likelihood)/N equal to 100. The smaller the normalized cost, the better the model describes the data. |
